# Supplementary material for: Analysis of Yes-Associated Protein-1 (YAP1) Target Gene Signature to Predict Progressive Breast Cancer
Source: J Clin Med. 2022 Mar 31;11(7):1947. doi: 10.3390/jcm11071947 (PMC8999906; doi:10.3390/jcm11071947)

Supplementary Figure 1: YAP1 expression and its association with overall survival of breast cancer patients

Kaplan-Meier survival curves are plotted for overall over eight years of follow-up for YAP1 expression (Quartile) for IDC (A) and subtypes (B-D) cohorts from METABRIC and TCGA datasets.

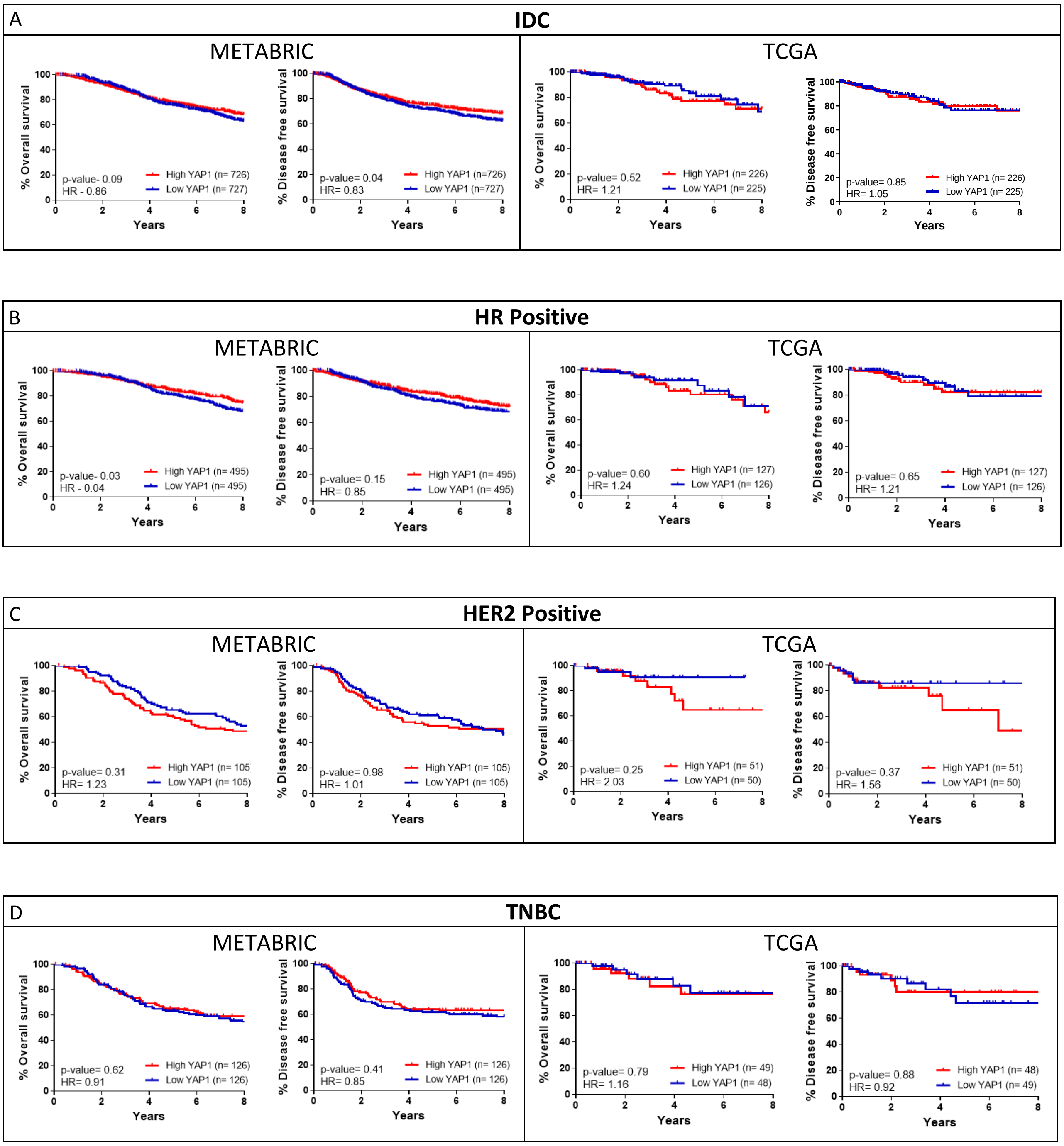

Supplementary Figure 2: Correlation of YAP1 target genes with each other for their expression in subtype cohorts

Gene expression of YAP1 targets correlated with each other for their expression in breast cancer tumors analyzed within IDC (A) and ER positive (B) subtypes.

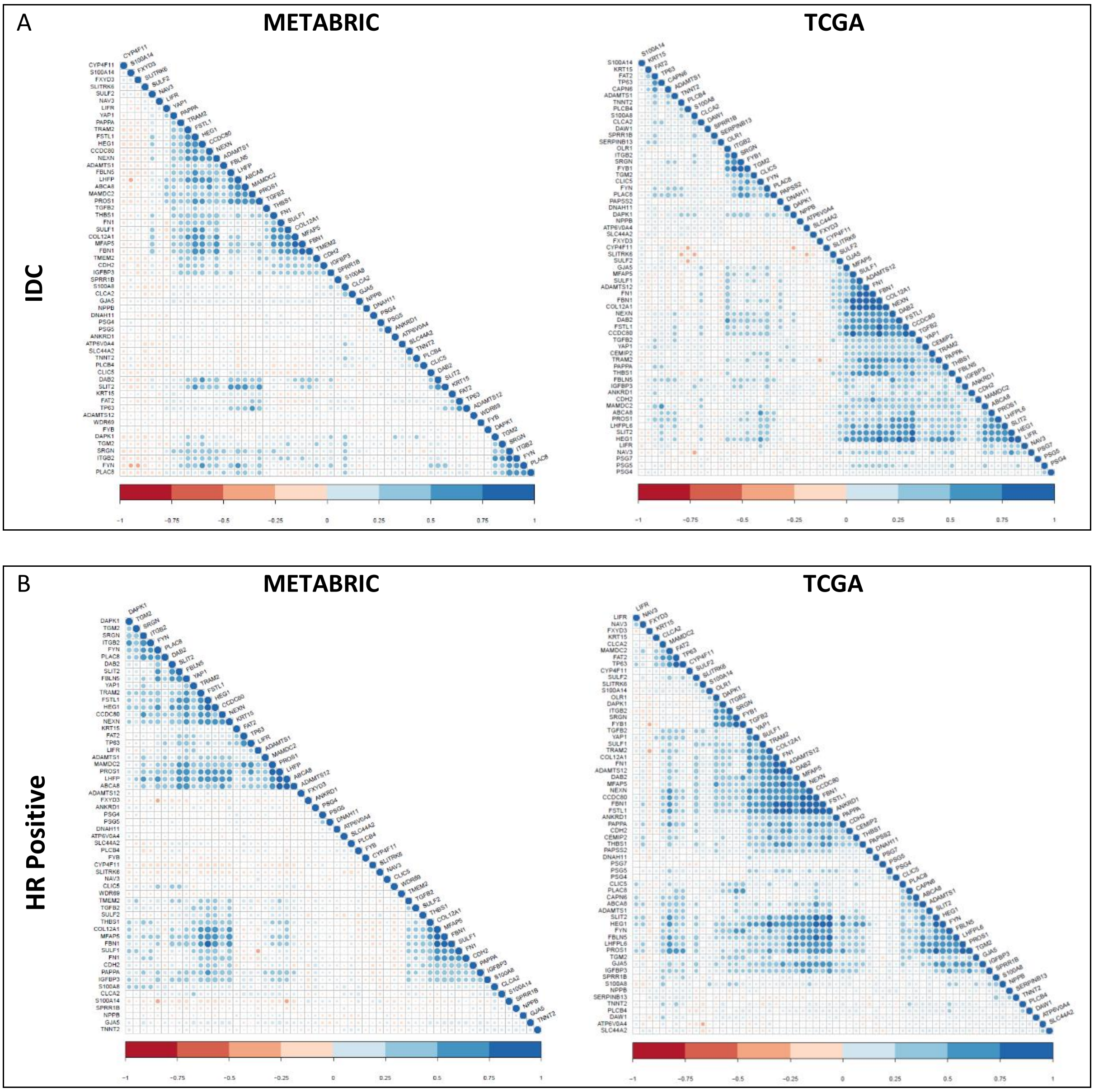

Supplement: Supplementary file 1 [file jcm-11-01947-s001.zip › Manuscript_Supplementary_Figures_GomathiV_et.al..pdf]
